# Investigation of Workability and Mechanical Properties of PVA Fiber-Reinforced Phosphogypsum-Based Composite Materials

**DOI:** 10.3390/ma16124244

**Published:** 2023-06-08

**Authors:** Ronggui Huang, Zhong Tao, Lei Wu, Jinjin Shen, Weijie Xu

**Affiliations:** 1 Faculty of Civil Engineering and Architecture, Kunming University of Science and Technology, Kunming 650599, China; 20212210030@stu.kust.edu.cn (R.H.); wulei0324@stu.kust.edu.cn (L.W.); 20222210005@stu.kust.edu.cn (J.S.); 20222210001@stu.kust.edu.cn (W.X.); 2 Earthquake Engineering Researching Center of Yunnan, Kunming 650599, China

**Keywords:** phosphogypsum-based construction material, PVA fiber, workability, mechanical properties, SEM analysis

## Abstract

To address the poor characteristics of low strength and poor toughness in phosphogypsum-based construction material, this study investigates the influence of different diameters, lengths, and dosages of polyvinyl alcohol (abbreviated as PVA) fibers on the workability and mechanical properties of phosphogypsum-based construction material. The results show that as the length and dosage of PVA fibers increase, the flowability of the slurry gradually decreases, and the setting time also shortens. With an increase in the diameter of PVA fibers, the rate of decrease in flowability slows down, and the rate of shortening of setting time also gradually slows down. Moreover, the inclusion of PVA fibers significantly improves the mechanical strength of the specimens. When PVA fibers with a diameter of 15 μm, length of 12 mm, and dosage of 1.6% are used, the phosphogypsum-based construction material reinforced with PVA fibers exhibits optimal performance. Under this mixing ratio, the strength values of the specimens for flexural strength, bending strength, compressive strength, and tensile strength are 10.07 MPa, 10.73 MPa, 13.25 MPa, and 2.89 MPa, respectively. Compared to the control group, the strength enhancements are 273.00%, 164.29%, 15.32%, and 99.31%, respectively. SEM scanning of the microstructure provides a preliminary explanation for the mechanism of how PVA fibers affect the workability and mechanical properties of phosphogypsum-based construction material. The findings of this study can provide a reference for the research and application of fiber-reinforced phosphogypsum-based construction material.

## 1. Introduction

Phosphogypsum, a prevalent solid waste product with large stockpiles and severe environmental pollution, is one of the most common bulk solid waste products currently [1,2,3,4]. China produces a huge amount of phosphogypsum annually, with an average annual emission of over 80 million tons from 2020 to 2022, but the utilization rate is less than 50%. According to China Building Materials News, the current stockpile of phosphogypsum in China is about 600 million tons, and the storage capacity of phosphogypsum sites is nearing saturation. The emission and stockpile of phosphogypsum are increasing year by year, which not only occupies a large amount of land resources but also causes severe pollution to water sources in nature due to the presence of free phosphorus, fluoride, and other elements, leading to ecological problems [5,6]. Utilizing phosphogypsum in the production of phosphogypsum-based construction materials and its application in the construction industry is a crucial approach to address the resource utilization of phosphogypsum [7,8]. However, the poor toughness and low strength of phosphogypsum-based construction materials hinder the effective implementation of this approach [9,10,11].

In order to enhance the toughness and strength of construction gypsum, numerous scholars both domestically and internationally have conducted extensive research, discovering that the incorporation of fibers can effectively improve the mechanical and physical properties of composite materials. At present, most researchers have primarily investigated the reinforcing effects of polypropylene fibers [11,12,13,14,15,16], glass fibers [17,18,19,20,21,22], basalt fibers [18,23,24,25,26], and plant fibers [27,28,29] on various types of construction gypsum. PVA fibers, a type of synthetic organic fiber with remarkable properties such as high strength and good toughness, are widely employed in the preparation of ECC materials. However, research on the impact of PVA fibers on the workability and mechanical properties of phosphogypsum-based construction materials is limited. Zhu [30] and colleagues examined the influence of PVA and PP fibers on the flexural strength and toughness of gypsum-based composites, finding that compared to PP fibers, PVA fibers significantly reduced the workability of hardened gypsum-based composites, accelerated the hydration process, and increased flexural strength and toughness. Li [18] et al. investigated the effects of BF, GF, and PVA fibers on the setting time, fluidity, water absorption rate, and flexural strength of gypsum-based composites. Their research revealed that as fiber length and volume increased, setting time and fluidity decreased, water absorption rate increased, and flexural strength improved by over 50%. Thus, it can be concluded that the incorporation of PVA fibers effectively enhances the mechanical and physical properties of gypsum-based composites. However, most current studies primarily focus on the influence of fiber length and dosage on the workability and mechanical properties of composites, while fiber diameter is also an essential factor that has been scarcely reported in the phosphogypsum construction field. Moreover, most researchers predominantly concentrate on the compressive and flexural strengths of composite materials to evaluate their mechanical performance, while tensile strength is also a crucial indicator for assessing the mechanical properties of fiber-reinforced composites, offering a comprehensive reflection of a composite material’s plastic deformation performance [31,32,33,34]. Nevertheless, this parameter has received limited attention in the phosphogypsum construction field.

In this study, PVA fiber-reinforced phosphogypsum-based composite materials (abbreviated as PVAEGC) were prepared with fiber diameters of 15 μm, 19 μm, and 31 μm; lengths of 3 mm, 6 mm, 9 mm, and 12 mm; and volume dosages of 0.4%, 0.8%, 1.2%, 1.6%, and 2.0%. The effects of PVA fiber diameter, length, and dosage on the workability and mechanical properties of phosphogypsum-based composites were assessed. Additionally, the dispersion uniformity of PVA fibers in phosphogypsum-based construction materials was evaluated through SEM scanning, providing a preliminary explanation of the mechanisms underlying the influence of PVA fibers on phosphogypsum construction material performance. The findings of this study can provide a reference for the research and application of fiber-reinforced phosphogypsum-based construction material.

## 2. Experiment

### 2.1. Raw Materials

#### 2.1.1. Phosphogypsum-Based Construction Material

**Figure 1:**
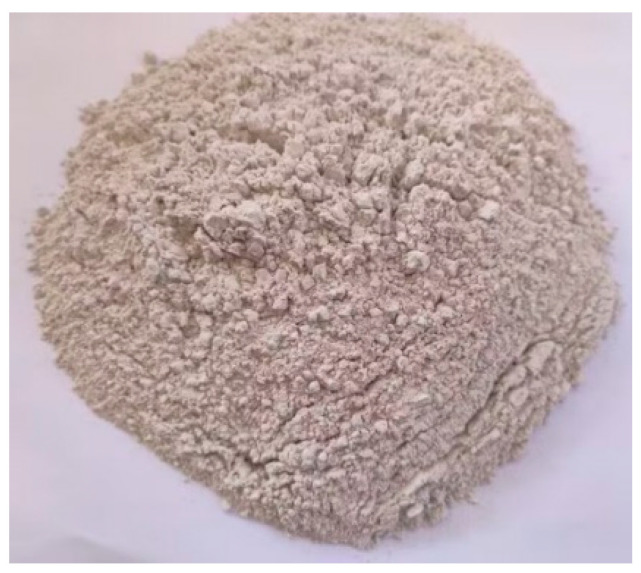
Phosphogypsum-based Construction Material.

The phosphogypsum-based construction material used in this study was provided by Yunnan Xuangan Environmental ProtectionTechnology Co., Ltd. (Kunming, China), After a series of pretreatment, such as impurity removal and neutralization, phosphogypsum raw materials are dehydrated at 140 °C for 6 to 8 h in an electric blast drying oven to produce phosphogypsum-based construction, as shown in Figure 1, and the XRF analysis results in Table 1.

#### 2.1.2. PVA Fibers

The PVA fibers used in this study were produced by Jiangsu Tianyi Engineering Fiber Co., Ltd. (Changzhou, China), with their physical and mechanical properties detailed in Table 2. The PVA fibers used in the experiment had lengths of 3 mm, 6 mm, 9 mm, and 12 mm, and diameters of 15 μm, 19 μm, and 31 μm.

### 2.2. Mix Proportion Design

**Figure 2:**
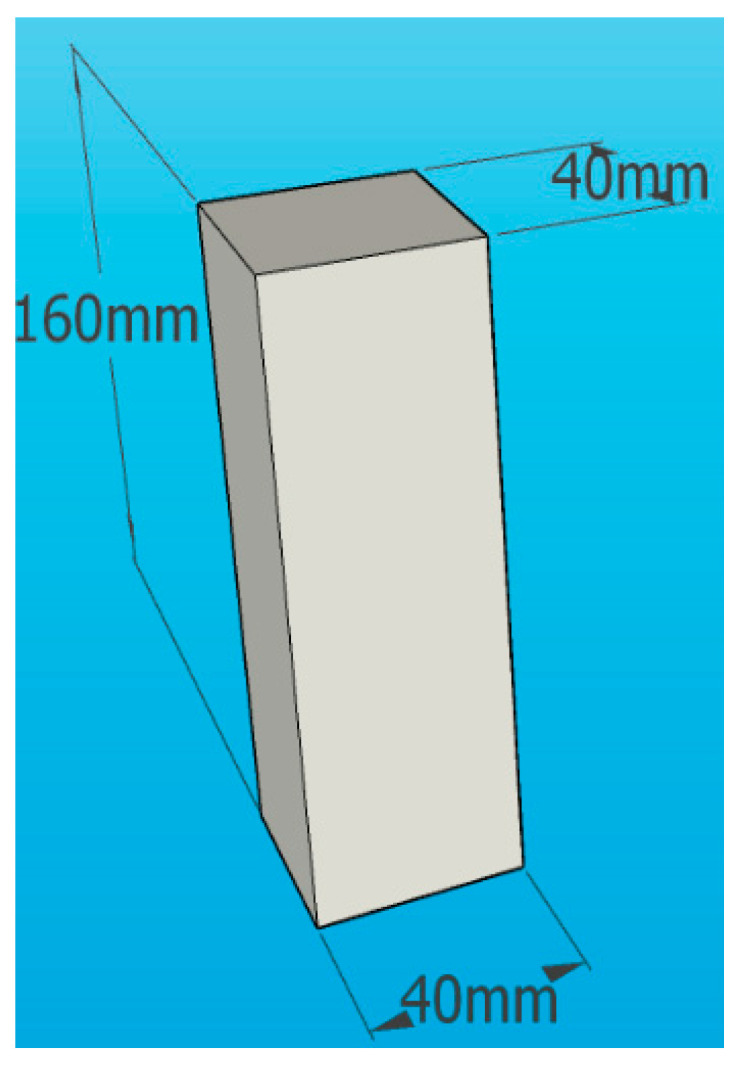
Cubic specimens.

**Figure 3:**
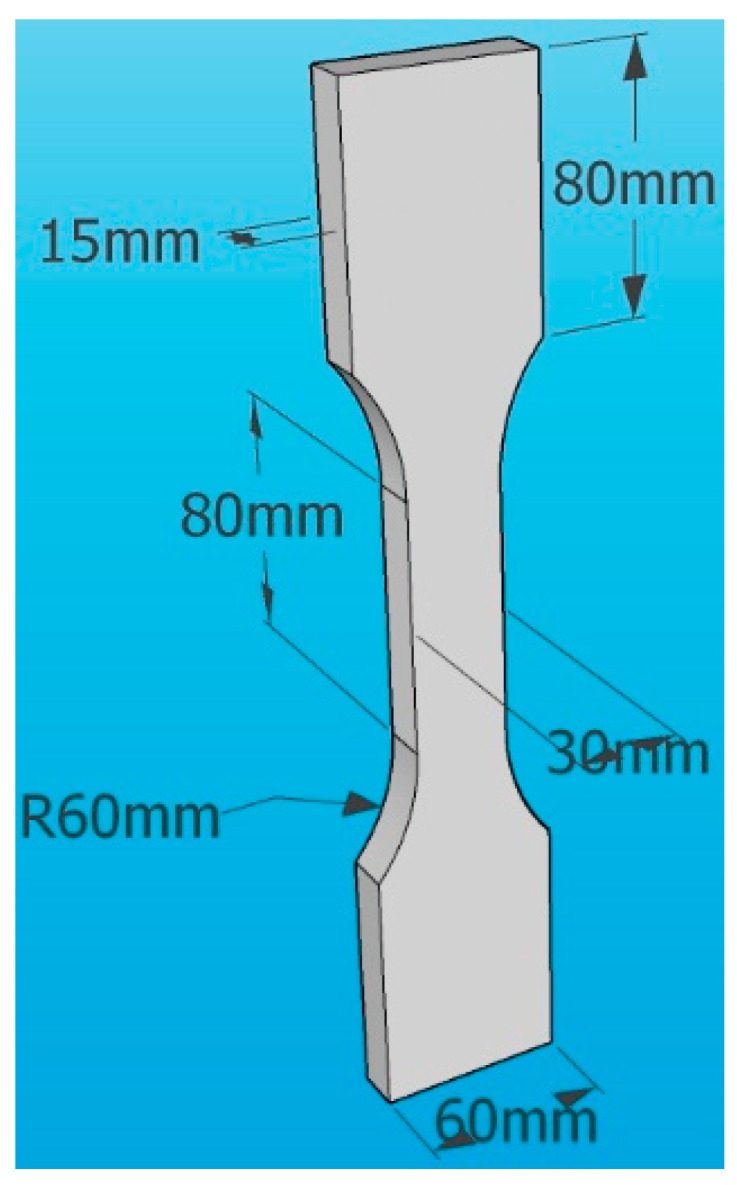
“Dog-bone” shaped specimen.

In this study, three commonly used diameters and four different lengths of PVA fibers in engineering applications were selected as the research objects. The fiber volume fraction was determined based on the experience of other scholars [8], and the water dosage was determined using a standard consistency test, as shown in Table 3. For each mixing ratio, six cubic specimens with dimensions of 40 mm × 40 mm × 160 mm (as shown in Figure 2) and three dog-bone-shaped specimens (as shown in Figure 3) were cast in the experiment. In total, 366 cubic specimens and 183 dog-bone-shaped specimens were cast in this study.

### 2.3. Experimental Test Methods

**Figure 4:**
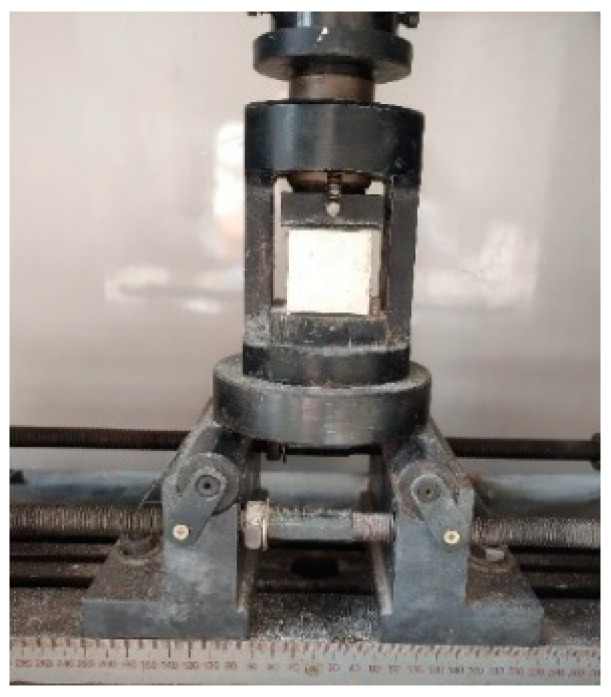
Compressive test loading apparatus.

**Figure 5:**
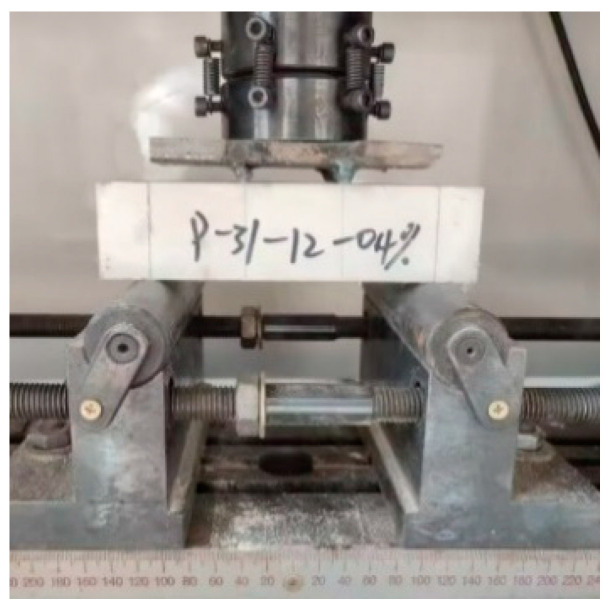
Flexural test loading apparatus.

**Figure 6:**
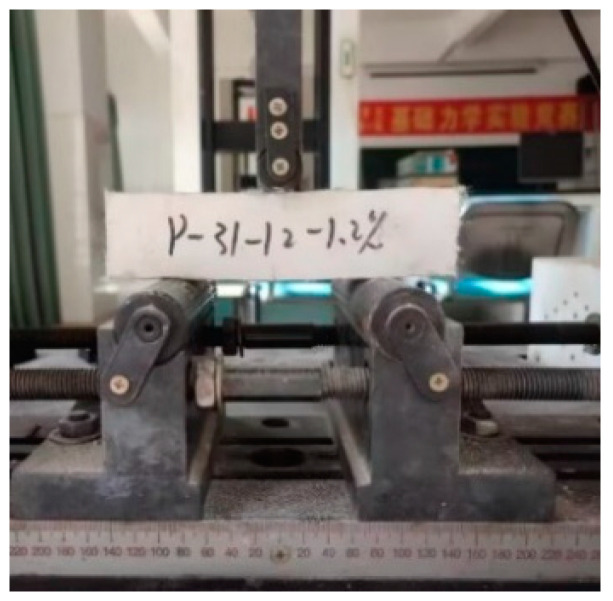
Bending test loading apparatus.

The fluidity and setting time of the PVAEGC slurry were measured according to the method specified in “Measurement of Physical Properties of Construction Gypsum Paste” (GB/T 17669.4-1999) [35]. The compressive, flexural, and bending strengths of PVAEGC were measured according to “Measurement of Mechanical Properties of Construction Gypsum” (GB/T 17669.3-1999) [36]. The loading devices are shown in Figure 4, Figure 5 and Figure 6, respectively.

**Figure 7:**
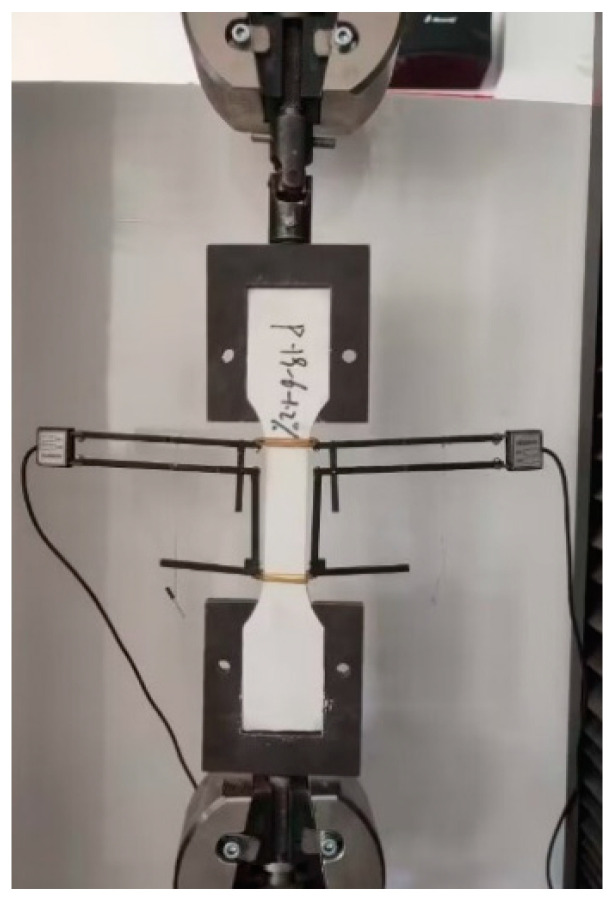
Tensile test loading apparatus.

The tensile strength of PVAEGC was measured using “dog-bone” shaped specimens, with detailed dimensions shown in Figure 3. A universal testing machine was used for tensile testing, and the loading device is illustrated in Figure 7. The loading rate was 0.15 mm/min, controlled by displacement, with a sampling frequency of 10 Hz.

The microscopic morphology of the PVAEGC cross-section was observed using a scanning electron microscope (SEM). Prior to testing, the specimen surfaces were coated with gold. The equipment model used was VEGA3.

## 3. Results and Discussion

### 3.1. The Influence of PVA Fibers on the Working Properties of PVAEGC

#### 3.1.1. The Influence of PVA Fibers on the Fluidity of Phosphogypsum-Based Construction Material

**Figure 8:**
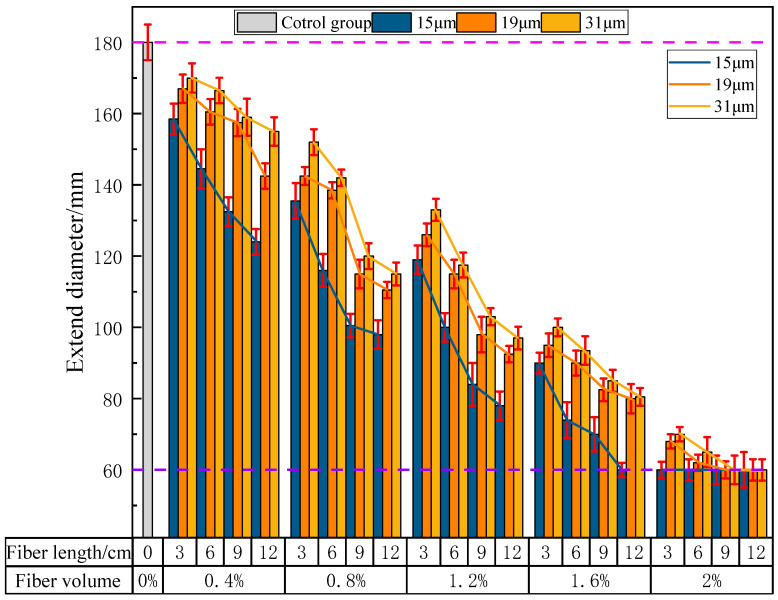
The influence of PVA fibers on the fluidity of PVAEGC.

Figure 8 shows the effect of PVA fibers on the fluidity of PVAEGC slurry. In the figure, the fluidity value of 60 mm indicates that the fluidity of the PVAEGC slurry cannot be measured. It can be seen from the figure that, with constant PVA fiber content and diameter, the fluidity of the PVAEGC slurry decreases as the length of the PVA fibers increases; with constant PVA fiber length and diameter, the fluidity of the PVAEGC slurry decreases as the PVA fiber content increases; with constant PVA fiber length and content, the decrease in the fluidity of the PVAEGC slurry gradually slows down as the diameter of the PVA fibers increases.

In the case of a PVA fiber diameter of 15 μm, when the fiber content reaches 1.6%, the slurry of the experimental group with a fiber length of 12 mm loses its fluidity. When the fiber content reaches 2.0%, the slurry of all experimental groups with different fiber lengths loses its fluidity. In the case of a PVA fiber diameter of 19 μm, when the fiber content reaches 2.0%, the slurry of the experimental groups with fiber lengths of 9 mm and 12 mm loses its fluidity. In the case of a PVA fiber diameter of 31 μm, when the fiber content reaches 2.0%, the slurry of the experimental groups with fiber lengths of 9 mm and 12 mm loses its fluidity.

#### 3.1.2. The Influence of PVA Fibers on the Setting Time of Phosphogypsum-Based Construction Material

(1) The influence of PVA fibers on the initial setting time of PVAEGC

**Figure 9:**
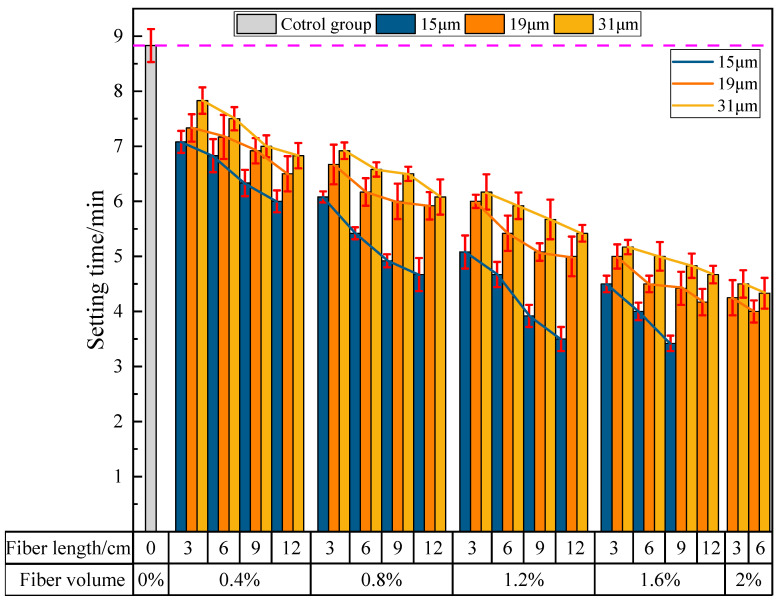
The influence of PVA fibers on the initial setting time of PVAEGC.

Figure 9 shows the effect of PVA fibers on the initial setting time of PVAEGC. For the experimental groups with slurry losing fluidity (the fluidity value is 60 mm in the figure), the setting time measurement will no longer be carried out. It can be seen from the figure that, with constant PVA fiber content and diameter, the initial setting time of PVAEGC slurry gradually shortens as the length of PVA fibers increases; with constant PVA fiber length and diameter, the initial setting time of PVAEGC slurry gradually shortens as the PVA fiber content increases; with constant PVA fiber length and content, the shortening speed of the initial setting time of PVAEGC slurry gradually slows down as the diameter of the PVA fibers increases.

For PVA fibers with a diameter of 15 μm, when the fiber content reaches 1.6% and the length reaches 9 mm, the initial setting time of the slurry is shortened from 8 min 50 s of the blank group to 3 min 25 s. For PVA fibers with a diameter of 19 μm, when the fiber content reaches 2.0% and the length reaches 6 mm, the initial setting time of the slurry is shortened from 8 min 50 s of the blank group to 4 min. For PVA fibers with a diameter of 31 μm, when the fiber content reaches 2.0% and the length reaches 6 mm, the initial setting time of the slurry is shortened from 8 min 50 s of the blank group to 4 min 33 s.

(2) The influence of PVA fibers on the final setting time of PVAEGC

**Figure 10:**
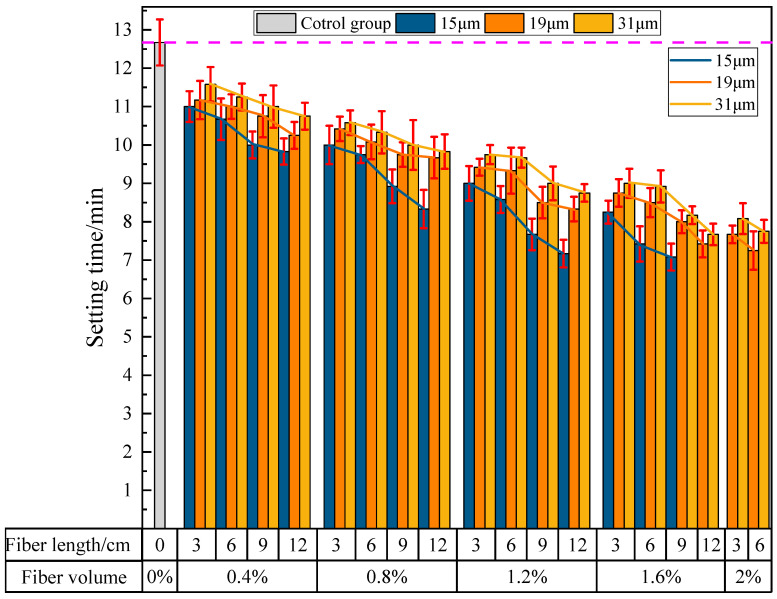
The influence of PVA fibers on the final setting time of PVAEGC.

Figure 10 shows the effect of PVA fibers on the final setting time of PVAEGC. From the figure, with constant PVA fiber content and diameter, the final setting time of PVAEGC slurry gradually shortens as the length of PVA fibers increases; with constant PVA fiber length and diameter, the final setting time of PVAEGC slurry gradually shortens as the PVA fiber content increases; with constant PVA fiber length and content, the shortening speed of the final setting time of PVAEGC slurry gradually slows down as the diameter of the PVA fibers increases.

For PVA fibers with a diameter of 15 μm, when the fiber content reaches 1.6% and the length reaches 9 mm, the final setting time of the slurry is shortened from 12 min 40 s of the blank group to 7 min 5 s. For PVA fibers with a diameter of 19 μm, when the fiber content reaches 2.0% and the length reaches 6 mm, the final setting time of the slurry is shortened from 12 min 40 s of the blank group to 7 min 15 s. For PVA fibers with a diameter of 31 μm, when the fiber content reaches 2.0% and the length reaches 6 mm, the final setting time of the slurry is shortened from 12 min 40 s of the blank group to 7 min 45 s.

### 3.2. The Influence of PVA Fibers on the Mechanical Properties of PVAEGC

#### 3.2.1. The Influence of PVA Fibers on the Flexural Strength of PVAEGC

**Figure 11:**
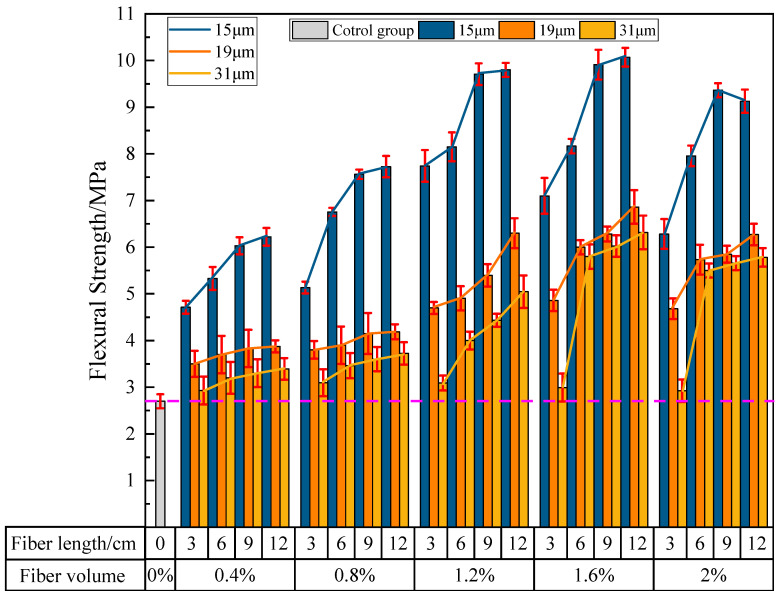
The influence of PVA fibers on the flexural strength of PVAEGC.

Figure 11 shows the effects of PVA fiber length, content, and diameter on the flexural strength of PVAEGC. From the figure, it can be seen that, with constant PVA fiber content and diameter, as the length of PVA fibers increases, the flexural strength of PVAEGC specimens generally shows a gradually increasing trend; with constant PVA fiber length and diameter, as the PVA fiber content increases, the flexural strength of PVAEGC specimens shows a trend of increasing first and then decreasing; with constant PVA fiber length and content, as the diameter of PVA fibers increases, the rate of the increase in flexural strength of PVAEGC specimens gradually slows down.

Under the condition of PVA fiber diameter being 15 μm, when the fiber content is 1.6% and the length is 12 mm, the flexural strength of the specimen reaches its maximum value, with a strength value of 10.07 MPa, an increase of 273.00% compared to the blank group. Under the condition of PVA fiber diameter being 19 μm, when the fiber content is 1.6% and the length is 12 mm, the flexural strength of the specimen reaches its maximum value, with a strength value of 6.86 Mpa, an increase of 154.11% compared to the blank group. Under the condition of PVA fiber diameter being 31 μm, when the fiber content is 1.6% and the length is 12 mm, the flexural strength of the specimen reaches its maximum value, with a strength value of 6.32 Mpa, an increase of 133.93% compared to the blank group.

In summary, the incorporation of PVA fibers can significantly improve the flexural strength of the specimens. The flexural strength of PVAEGC specimens is optimal when the diameter of PVA fibers is 15 μm, the content is 1.6%, and the length is 12 mm.

#### 3.2.2. The Influence of PVA Fibers on the Compressive Strength of PVAEGC

**Figure 12:**
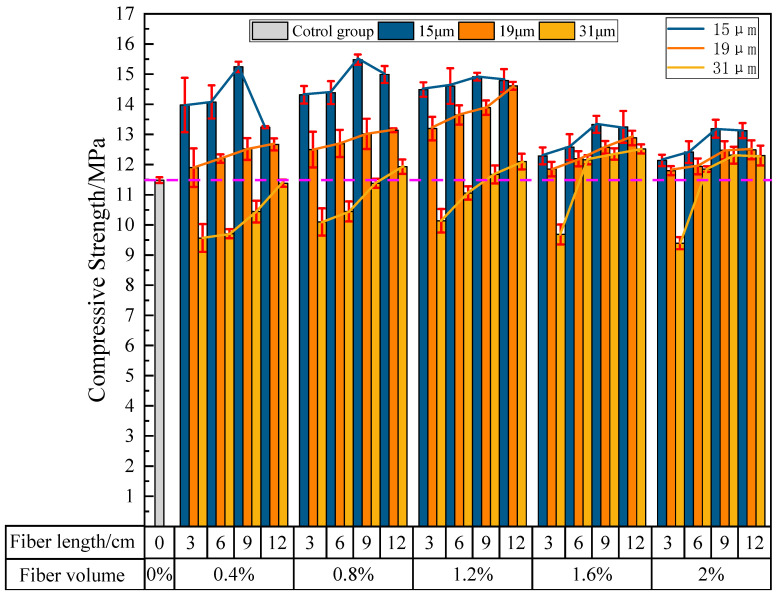
The influence of PVA fibers on the compressive strength of PVAEGC.

Figure 12 shows the effects of PVA fiber length, content, and diameter on the compressive strength of PVAEGC. With constant PVA fiber content and diameter, as the length of PVA fibers increases, the compressive strength of PVAEGC specimens generally shows a gradually increasing trend; with constant PVA fiber length and diameter, as the PVA fiber content increases, the compressive strength of PVAEGC specimens shows a trend of increasing first and then decreasing; with constant PVA fiber length and content, as the diameter of PVA fibers increases, the rate of the increase in compressive strength of PVAEGC specimens gradually slows down, and when the fiber diameter is too large and the content is small, the compressive strength of the specimens is lower than that of the blank group.

Under the condition of the PVA fiber diameter being 15 μm, when the fiber content is 0.8% and the length is 9 mm, the compressive strength of the specimen reaches its maximum value, with a strength value of 15.48 MPa, an increase of 34.73% compared to the blank group. Under the condition of the PVA fiber diameter being 19 μm, when the fiber content is 1.2% and the length is 12 mm, the compressive strength of the specimen reaches its maximum value, with a strength value of 14.61 MPa, an increase of 27.15% compared to the blank group. Under the condition of the PVA fiber diameter being 31 μm, when the fiber content is 1.6% and the length is 12 mm, the compressive strength of the specimen reaches its maximum value, with a strength value of 12.52 MPa, an increase of 8.96% compared to the blank group.

In summary, the influence of PVA fibers on the compressive strength of PVAEGC is relatively small. The compressive strength of the specimens is optimal when the diameter of PVA fibers is 15 μm, the content is 0.8%, and the length is 9 mm.

#### 3.2.3. The Influence of PVA Fibers on the Bending Strength of Phosphogypsum-Based Building Materials

**Figure 13:**
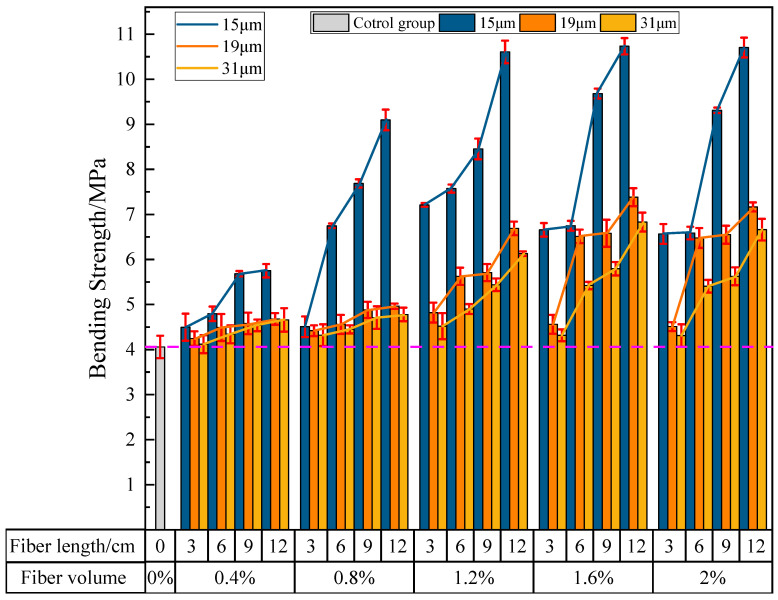
The influence of PVA fibers on the bending strength of PVAEGC.

Figure 13 shows the effects of PVA fiber length, content, and diameter on the bending strength of PVAEGC. With constant PVA fiber content and diameter, as the length of PVA fibers increases, the bending strength of PVAEGC specimens gradually increases; with constant PVA fiber length and diameter, as the PVA fiber content increases, the bending strength of PVAEGC specimens shows a trend of increasing first and then decreasing; with constant PVA fiber length and content, as the diameter of PVA fibers increases, the rate of the increase in bending strength of PVAEGC specimens gradually slows down.

Under the condition of the PVA fiber diameter being 15 μm, when the fiber content is 1.6% and the length is 12 mm, the bending strength of the specimen reaches its maximum value, with a strength value of 10.73 MPa, an increase of 164.29% compared to the blank group. Under the condition of the PVA fiber diameter being 19 μm, when the fiber content is 1.6% and the length is 12 mm, the bending strength of the specimen reaches its maximum value, with a strength value of 7.38 MPa, an increase of 81.77% compared to the blank group. Under the condition of the PVA fiber diameter being 31 μm, when the fiber content is 1.6% and the length is 12 mm, the bending strength of the specimen reaches its maximum value, with a strength value of 6.83 MPa, an increase of 68.23% compared to the blank group.

In summary, PVA fibers can significantly improve the bending strength of PVAEGC. The bending strength of the specimens is optimal when the diameter of PVA fibers is 15 μm, the content is 1.6%, and the length is 12 mm.

#### 3.2.4. The Influence of PVA Fibers on the Tensile Strength of PVAEGC

**Figure 14:**
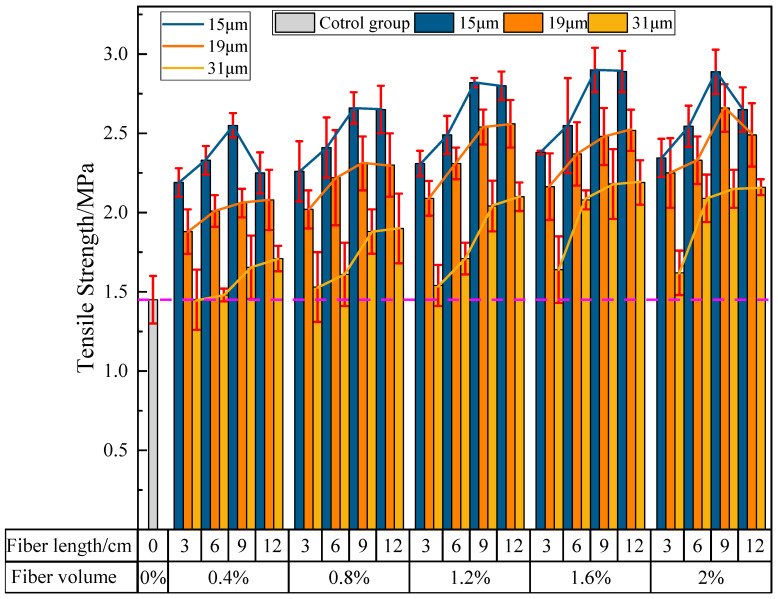
The influence of PVA fibers on the tensile strength of PVAEGC.

Figure 14 shows the effects of PVA fiber length, content, and diameter on the tensile strength of PVAEGC. With constant PVA fiber content and diameter, as the length of PVA fibers increases, the tensile strength of PVAEGC specimens generally shows a gradually increasing trend; with constant PVA fiber length and diameter, as the PVA fiber content increases, the tensile strength of PVAEGC specimens presents a trend of increasing first and then decreasing; with constant PVA fiber length and content, as the diameter of PVA fibers increases, the rate of the increase in tensile strength of PVAEGC specimens gradually slows down.

Under the condition of the PVA fiber diameter being 15 μm, when the fiber content is 1.6% and the length is 9 mm, the tensile strength of the specimen reaches its maximum value, with a strength value of 2.90 MPa, an increase of 100.00% compared to the blank group. Under the condition of the PVA fiber diameter being 19 μm, when the fiber content is 1.6% and the length is 9 mm, the tensile strength of the specimen reaches its maximum value, with a strength value of 2.67 MPa, an increase of 84.14% compared to the blank group. Under the condition of the PVA fiber diameter being 31 μm, when the fiber content is 1.6% and the length is 12 mm, the tensile strength of the specimen reaches its maximum value, with a strength value of 2.19 MPa, an increase of 51.03% compared to the blank group.

In summary, PVA fibers can significantly improve the tensile strength of PVAEGC. The tensile strength of the specimens is optimal when the diameter of PVA fibers is 15 μm, the content is 1.6%, and the length is 9 mm.

#### 3.2.5. The Influence of PVA Fibers on the Flexural-to-Compressive Strength Ratio and Tensile-to-Compressive Strength Ratio of PVAEGC

**Figure 15:**
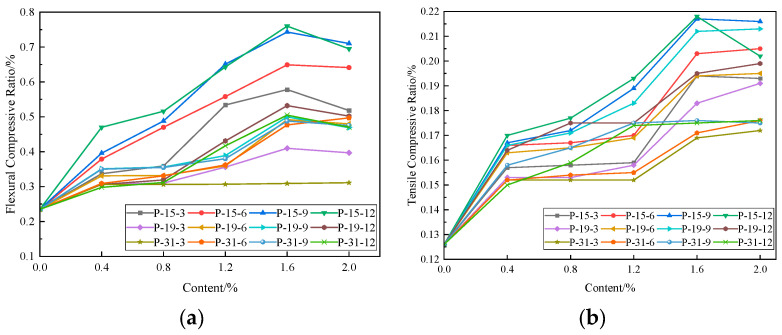
The influence of PVA fibers on the flexural-to-compressive strength ratio and tensile-to-compressive strength ratio of PVAEGC: (**a**) flexural-to-compressive strength ratio and (**b**) tensile-to-compressive strength ratio.

The flexural-to-compressive strength ratio and tensile-to-compressive strength ratio are important indicators for evaluating the toughness of composite materials. The higher the ratios, the better the toughness of the composite material, and vice versa. Figure 15 shows the influence of PVA fibers on the tensile-to-compressive strength ratio and flexural-to-compressive strength ratio of PVAEGC. In the legend, “P-15-3” represents the addition of PVA fibers with a diameter of 15 μm and a length of 3 mm, and the other legends follow the same pattern.

As shown in the figure, the addition of PVA fibers can significantly improve the flexural-to-compressive strength ratio and tensile-to-compressive strength ratio of PVAEGC. As the diameter of PVA fibers increases, both the flexural-to-compressive strength ratio and tensile-to-compressive strength ratio of PVAEGC show a downward trend, indicating that PVA fibers with smaller diameters can better improve the toughness of composite materials. When the fiber content does not exceed 1.6%, the flexural-to-compressive strength ratio and tensile-to-compressive strength ratio of PVAEGC show an increasing trend; when the fiber content continues to increase, the ratios of some specimens decrease.

When the diameter of PVA fibers is 15 μm, the length is 12 mm, and the content is 1.6%, both the tensile-to-compressive strength ratio and flexural-to-compressive strength ratio of PVAEGC reach their peak values. The peak values of the flexural-to-compressive strength ratio and tensile-to-compressive strength ratio are 0.760 and 0.218, respectively, which are 223.40% and 73.02% higher than those of the blank group, respectively.

#### 3.2.6. Potential Research Value and Application Prospect of PVAEGC

Due to the poor characteristics of low strength and poor toughness in phosphogypsum-based construction materials, they cannot be widely used. Many researchers have attempted to enhance the performance of phosphogypsum-based construction materials by incorporating various types of fibers. As shown in Table 4, compared to basalt fibers, carbon fibers, and polypropylene fibers, PVA fibers can significantly enhance the flexural strength and bending strength of phosphogypsum-based construction materials, with strength enhancements of 273.0% and 164.3%, respectively, compared to the control group. However, the enhancement effect on compressive strength is not as significant, with only a 30.5% increase, while polypropylene fibers achieve a 50.4% increase in compressive strength. Therefore, utilizing PVA fibers to prepare high-toughness PVAEGC and applying them in fields with high toughness requirements is an effective way to utilize phosphogypsum-based construction materials, and it holds promising application prospects.

### 3.3. Observation of the Microstructure of PVA Fibers and Analysis of Their Influence Mechanism on PVAEGC

Through SEM analysis of PVAEGC containing PVA fibers with different lengths and contents, the microstructure and bonding situation between fibers and the phosphogypsum-based matrix under different influencing factors were obtained, and a preliminary analysis of the influence mechanism was conducted based on the above experimental results. Due to the small range that can be observed in SEM scanning, it is not possible to distinguish the changes in fiber length.

#### 3.3.1. The Influence Mechanism of PVA Fibers on the Workability of PVAEGC

**Figure 16:**
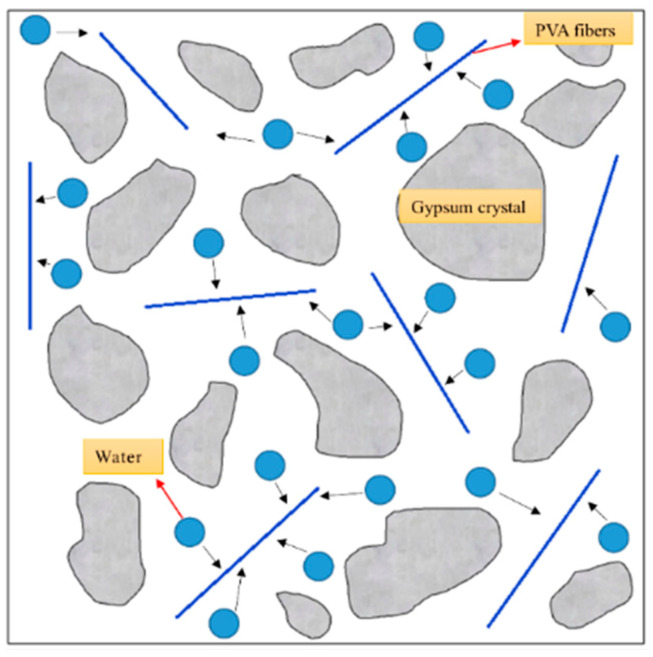
Model of the influence of PVA fibers on the distribution of free water in PVAEGC slurry.

As the length and content of PVA fibers increase, a three-dimensional network structure is formed in the slurry, which increases the internal friction of the slurry and leads to a decrease in fluidity. In addition, as shown in Figure 16, the molecular structure of PVA fibers contains hydroxyl groups, which are hydrophilic groups that can adsorb a part of free water, causing a change in the water distribution in the slurry and thus making the PVAEGC slurry lose its plasticity earlier. The black arrow in the figure indicates the moving direction of water.

When the fiber length and content are constant, as the diameter of PVA fibers increases, the number of fibers decreases, and the specific surface area of the fibers gradually decreases. As a result, the amount of free water adsorbed by PVA fibers in the slurry also decreases, leading to a slower decrease in fluidity and an increase in setting time. In addition, when the fiber length and content are constant, fibers with smaller diameters have more roots, which makes it easier to form a three-dimensional network structure in the slurry, resulting in a faster decrease in the fluidity of the slurry.

#### 3.3.2. The Influence Mechanism of PVA Fibers on the Mechanical Properties of PVAEGC

**Figure 17:**
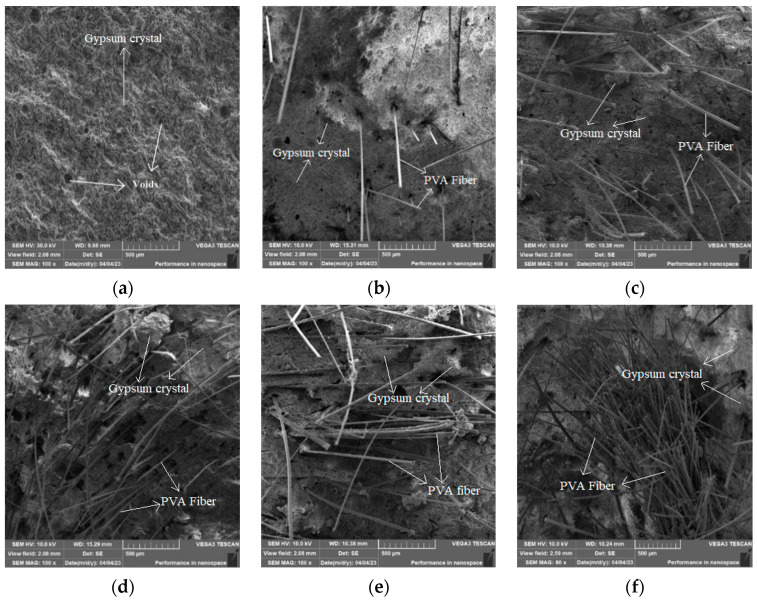
Microstructure under different PVA fiber contents (**a**) blank group, (**b**) 0.4%, (**c**) 0.8%, (**d**) 1.2%, (**e**) 1.6%, (**f**) 2.0%.

As can be seen from Figure 17a, the hardened phosphogypsum is a porous material and PVA fibers have good hydrophilicity which can be better combined with the phosphogypsum matrix, making the internal structure of PVAEGC more compact and thereby improving the strength of PVAEGC. In addition, the addition of PVA fibers can effectively transfer stress and play a good bridging role. The bridging effect of PVA fibers changes the internal stress distribution of PVAEGC, limits the extension of stress, and makes the specimen bear the external load together with the matrix, achieving a toughening effect and improving the flexural strength [24].

**Figure 18:**
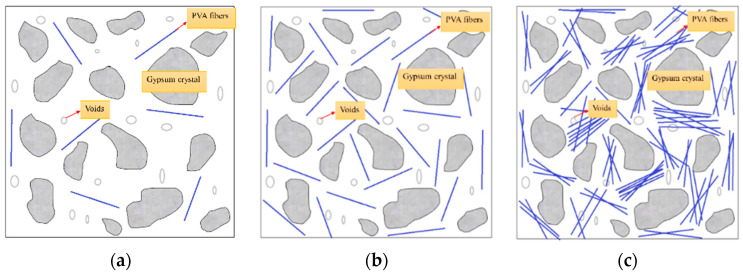
PVAEGC model under different PVA fiber contents: (**a**) too little content, (**b**) moderate content, and (**c**) excessive content.

From Figure 17 and Figure 18, it can be seen that when the content of PVA fibers is too low, the fibers cannot be completely and uniformly dispersed in the gypsum matrix, and the distance between the fibers is relatively large (as shown in Figure 17b and Figure 18a). Although the bridging effect occurs in the matrix under external force, the strength is improved but the crack restriction is not significant and the strength improvement is limited. When the content of PVA fibers is moderate, PVA fibers are evenly distributed in the matrix without entanglement or agglomeration (as shown in Figure 18b), and the hydrophilic hydroxyl groups in PVA fibers are conducive to the precipitation and crystallization of calcium sulfate dihydrate on their surface, resulting in better adhesion between fibers and phosphogypsum matrix, making the internal structure of PVAEGC more compact, and thus improving the bridging effect of fibers [37]. When the content of PVA fibers is excessive, the dispersion ability of PVA fibers in the slurry is poor, and it is prone to phenomena such as crossing, entanglement, and agglomeration of uneven distribution (as shown in Figure 17f and Figure 18c), which will increase the internal pores and defects of the specimens, leading to an increase in porosity and a decrease in the compactness of the matrix. After the slurry hardens, the content of phosphogypsum in these unevenly distributed areas is relatively small, becoming stress concentration areas, which leads to a decrease in the strength of PVAEGC [37].

**Figure 19:**
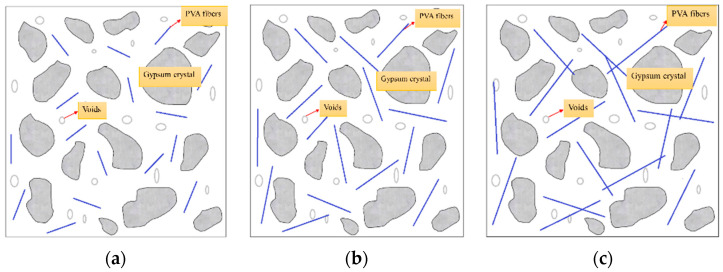
PVAEGC model under different PVA fiber lengths (**a**) short length, (**b**) medium length, (**c**) long length.

From Figure 19, it can be seen that when short PVA fibers are added, although they can play a certain bridging role, the length is too short (Figure 19a), making it easy for the fibers to be pulled out when PVAEGC cracks, so the strength improvement effect is not very significant. As the length increases, the bonding force between PVA fibers and the gypsum matrix hinders the pull-out of the fibers, thereby preventing the development of cracks and improving the strength. When the length of PVA fibers continues to increase, their dispersion ability in the slurry becomes worse, and it is prone to phenomena such as crossing, entanglement, and agglomeration of uneven distribution (as shown in Figure 19c). After the slurry hardens, the content of phosphogypsum in these unevenly distributed areas is relatively small, becoming stress concentration areas, which leads to a decrease in the strength of the specimens. In addition, when the length of PVA fiber is too short to reach the critical length of the fiber, the phosphorus building gypsum base may not be able to effectively transfer the load to the PVA fiber, resulting in low flexural strength of the specimen. When the length of PVA fiber is moderate and meets the critical length of fiber, PVA fiber can effectively share load and provide enough deformation capacity, which greatly improves the flexural strength of the specimen [39].

**Figure 20:**
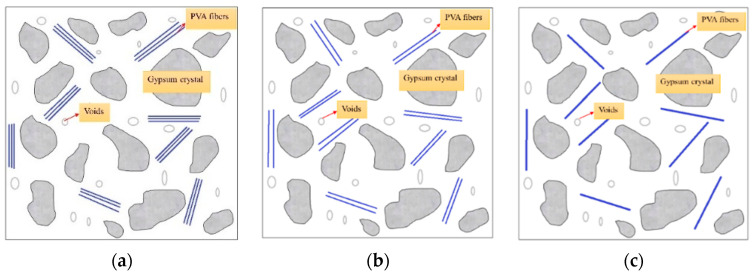
PVAEGC model under different PVA fiber diameters (**a**) small diameter, (**b**) medium diameter, (**c**) large diameter.

The mechanical strength of PVAEGC depends on the strength of the fiber bridging stress. From the perspective that the bridging stress of the fiber is a function of the fiber quantity, the finer the fiber, the more the number of fibers under the same content (as shown in Figure 20), which is more beneficial to the bridging stress, so the strength is higher. In addition, the strength of PVAEGC is also related to the wrapping force of the fibers [35]. Under the same content, the finer the fiber diameter, the greater the number of fibers, and the larger the specific surface area of the fibers, which increases the wrapping force of the fibers, thereby leading to an increase in the strength of PVAEGC [40,41].

## 4. Conclusions

This study evaluates the influence of PVA fibers with different lengths, diameters, and dosages on the workability and mechanical properties of phosphogypsum-based construction material. Based on the experimental results and analysis, the following conclusions can be drawn:

PVA fibers can reduce the fluidity of PVAEGC slurry. As the length and content of PVA fibers increase, the fluidity of the PVAEGC slurry gradually decreases. As the diame-ter of PVA fibers increases, the rate of decrease in the fluidity of PVAEGC slurry gradually slows down.

PVA fibers can shorten the setting time of PVAEGC slurry. As the length and content of PVA fibers increase, the initial and final setting times of the slurry gradually shorten. As the diameter of PVA fibers increases, the rate of shortening of the initial setting time of PVAEGC slurry gradually slows down.

PVA fibers can significantly improve the flexural strength, bending strength, and ten-sile strength of PVAEGC, but the improvement effect on flexural strength is not obvious. When PVA fibers with a diameter of 15 μm, length of 12 mm, and volume fraction of 1.6% are used, the specimens exhibit maximum values for flexural strength, bending strength, and tensile strength, with strength values of 10.071 MPa, 10.73 MPa, and 2.89 MPa, respectively. Compared to the control group, the strength enhancements are 273.00%, 164.29%, and 99.31%, respectively. When the fiber diameter is 15 μm, length is 12 mm, and volume fraction is 0.8%, the compressive strength of the specimens reaches a maximum of 14.99 MPa, with a strength enhancement of 30.46% compared to the control group.

Considering all performance aspects, the optimal performance of PVARGC is achieved when PVA fibers with a diameter of 15 μm, length of 12 mm, and dosage of 1.6% are used. Under this mixing ratio, the strength values of the specimens for flexural strength, bending strength, compressive strength, and tensile strength are 10.07 MPa, 10.73 MPa, 13.25 MPa, and 2.89 MPa, respectively. Compared to the control group, the strength enhancements are 273.00%, 164.29%, 15.32%, and 99.31%, respectively.

Based on the experimental results and analysis, it is recommended that engineers and designers consider the cost-effectiveness of PVAEGC and conduct comprehensive cost-benefit analyses in specific projects, while balancing cost and performance. Additionally, it is suggested to conduct more tests in different environments and conditions to ensure that the optimal mixing ratio provided by the experimental results maintains superior performance in various situations.

## Figures and Tables

**Table 1:** XRF analysis.

Component	SiO_2_	Al_2_O_3_	Fe_2_O_3_	MnO	CaO	K_2_O	P_2_O_5_	SO_3_	S_r_O
Content (%)	6.758	0.395	0.052	0.102	37.993	0.082	1.021	53.524	0.073

**Table 2:** Physical properties of PVA fibers.

Fiber Type	Monofilament Diameter/μm	Elongation/%	Tensile Strength/MPa	Elastic Modulus/GPa	Density/g/cm^3^
PVA fiber	15	6.9	1830	40	1.29
	19	10	1660	40	1.3
	31	14	1500	38	1.3

**Table 3:** Mix Proportion Design.

Type	Fiber Length/mm	Fiber Length/%	Monofilament Diameter/μm	Water/g	Phosphogypsum-Based Construction Material/g
Control group	-	0	-	195	300
P-15/19/31-3-0.4%	3	0.4	15/19/31		
P-15/19/31-3-0.8%		0.8			
P-15/19/31-3-1.2%		1.2			
P-15/19/31-3-1.6%		1.6			
P-15/19/31-3-2.0%		2.0			
P-15/19/31-6-0.4%	6	0.4			
P-15/19/31-6-0.8%		0.8			
P-15/19/31-6-1.2%		1.2			
P-15/19/31-6-1.6%		1.6			
P-15/19/31-6-2.0%		2.0			
P-15/19/31-9-0.4%	9	0.4			
P-15/19/31-9-0.8%		0.8			
P-15/19/31-9-1.2%		1.2			
P-15/19/31-9-1.6%		1.6			
P-15/19/31-9-2.0%		2.0			
P-15/19/31-12-0.4%	12	0.4			
P-15/19/31-12-0.8%		0.8			
P-15/19/31-12-1.2%		1.2			
P-15/19/31-12-1.6%		1.6			
P-15/19/31-12-2.0%		2.0			

Note: “P-15/19/31-3-0.4%” indicates that under the condition of maintaining the length of PVA fibers at 3 mm and the volume fraction at 0.4%, PVA fibers with diameters of 15 μm, 19 μm, and 31 μm were selected for the experiment, and the remaining numbers are the same.

**Table 4:** Influence of PVA fiber incorporation and other fiber incorporation on phosphogypsum-based construction material.

Index	Here			Incorporating Basalt Fibers [24]			Incorporating Carbon Fibers [37]			Incorporating Polypropylene Fibers [38]		
	Control Group	Maximum Value	Amplitude Increase	Control Group	Maximum Value	Amplitude Increase	Control Group	Maximum Value	Amplitude Increase	Control Group	Maximum Value	Amplitude Increase
Flexural Strength/MPa	2.70	10.07	273.0%	-	-	-	4.50	7.90	75.6%	6.46	8.98	30.0%
Bending Strength/MPa	4.06	10.73	164.3%	4.37	7.78	78.2%	-	-	-	-	-	-
Compressive Strength/MPa	11.49	14.99	30.5%	34.65	38.35	10.7%	9.90	11.90	20.2%	17.63	26.52	50.4%
Tensile Strength/MPa	1.45	2.89	99.3%	-	-	-	-	-	-	-	-	-

## Data Availability

Data will be made available on request.

## References

[1] Li Y., Ding X., Guo Y., Rong C., Wang L., Qu Y., Ma X., Wang Z. (2011). A new method of comprehensive utilization of rice husk. J. Hazard. Mater..

[2] Rashad A.M. (2017). Phosphogypsum as a construction material. J. Clean. Prod..

[3] Zhang J., Xie W.M., Dong X.B., Yang H.M. (2023). Research progress on comprehensive utilization of phosphogypsum materials. Mater. Rep..

[4] Jia X.W., Wu Z., Ma Y. (2013). Resource utilization status of phosphogypsum building materials. Mater. Introd..

[5] Cui Y., Wang Q., Xue J. (2020). Novel Foam Insulation Material Produced by Calcined Phosphogypsum and H_2_O_2_. J. Mater. Civ. Eng..

[6] Yang M., Qian J.S., Wang Z., Huang Y.B. (2007). Effects of impurities on the application performance of phosphogypsum. Mater. Guide.

[7] Zhang L., Zhang A., Li K., Wang Q., Han Y., Yao B., Gao X., Feng L. (2020). Research on the pretreatment and mechanical performance of undisturbed phosphogypsum. Case Stud. Constr. Mater..

[8] Wu L. (2019). Study on Properties of Chopped Fiber-Phosphorus Building Gypsum Composites. Ph.D. Thesis.

[9] Vasconcelos G., Lourenço P.B., Camões A., Martins A., Cunha S. (2015). Evaluation of the performance of recycled textile fibres in the mechanical behaviour of a gypsum and cork composite material. Cem. Concr. Compos..

[10] Han S., Zhao Z.M., Cheng Y.H., Liu Y.C., Quan S.C. (2014). On Pretreatment Experimental Study of Yunnan Phosphorus Building Gypsum. Adv. Mater. Res..

[11] Deng Y., Furuno T. (2001). Properties of gypsum particleboard reinforced with polypropylene fibers. J. Wood Sci..

[12] Lu Y., Liu X., Lü K., Li Y., Liu F., Liu P. (2020). Properties and Fracture Surface Features of Plaster Mold Reinforced with Short Polypropylene Fibers for Investment Casting. Int. J. Met..

[13] Romero-Gómez M.I., Pedreño-Rojas M.A., Pérez-Galvez F., Rubio-de-Hita P. (2020). Characterization of gypsum composites with polypropylene fibers from non-degradable wet wipes. J. Build. Eng..

[14] Gencel O., del Coz Diaz J.J., Sutcu M., Koksal F., Rabanal F.A., Martinez-Barrera G., Brostow W. (2014). Properties of gypsum composites containing vermiculite and polypropylene fibers: Numerical and experimental results. Energy Build..

[15] Gencel O., del Coz Diaz J.J., Sutcu M., Koksal F., Rabanal F.P.Á., Martinez-Barrera G. (2016). A novel lightweight gypsum composite with diatomite and polypropylene fibers. Constr. Build. Mater..

[16] Yi Q., Chen H.N., Zeng Z., Wang Q.Y., Huang X.Z. (2018). Mechanical properties of polypropylene fiber reinforced phosphogypsum composites. J. Guizhou Univ. (Nat. Sci. Ed.).

[17] Li Z.X., Wang X., Yan W.L., Ding L., Liu J., Wu Z., Huang H. (2023). Physical and mechanical properties of gypsum-based composites reinforced with basalt, glass, and PVA fibers. J. Build. Eng..

[18] Gonçalves R.M., Martinho A., Oliveira J.P. (2022). Evaluating the potential use of recycled glass fibers for the development of gypsum-based composites. Constr. Build. Mater..

[19] Gouri K.S.R., Devdas M., Meher P.A. (2022). Lateral load behaviour of Glass Fibre Reinforced Gypsum walls supported on Reinforced Concrete frames. Structures.

[20] Rovero L., Galassi S., Misseri G. (2020). Experimental and analytical investigation of bond behavior in glass fiber-reinforced composites based on gypsum and cement matrices. Compos. Part B.

[21] Ngah S.A., Dams B., Ansell M.P., Stewart J., Hempstead R., Ball R.J. (2020). Structural performance of fibrous plaster. Part 1: Physical and mechanical properties of hessian and glass fibre reinforced gypsum composites. Constr. Build. Mater..

[22] Wang Q.Y., Sun J.M., Chen H.N., Liu Y., Ma K.J. (2017). Mechanical properties of glass fiber reinforced phosphogypsum composites. J. Guizhou Univ. (Nat. Sci. Ed.).

[23] Xie L., Zhou Y.S., Xiao S.H., Miao X., Murzataev A., Kong D., Wang L. (2022). Research on basalt fiber reinforced phosphogypsum-based composites based on single factor test and RSM test. Constr. Build. Mater..

[24] Li X., Yu T., Park S.J., Kim Y.H. (2022). Reinforcing effects of gypsum composite with basalt fiber and diatomite for improvement of high-temperature endurance. Constr. Build. Mater..

[25] Yildizel S.A. (2020). Material Properties of Basalt-Fiber-Reinforced Gypsum-Based Composites Made with Metakaolin and Silica Sand. Mech. Compos. Mater..

[26] Wu L., Zhao Z.M., Quan S.C., Wang C., Liu Z. (2019). Study on the effect of chopped fiber on the working performance of phosphorus building gypsum. Silic. Bull..

[27] Farmizan P., Asmahani A. (2022). Enhanced Mechanical Properties Plaster of Paris with Addition of Rice Husk Fibers. IOP Conf. Ser. Earth Environ. Sci..

[28] Babu K.S., Ratnam C. (2021). Mechanical and thermophysical behavior of hemp fiber reinforced gypsum composites. Mater. Today Proc..

[29] Zhang T.X., Liao Y.S., Liu L.J., Wang H.B., Dong Q. (2023). Study on water resistance and mechanical properties of ramie fiber reinforced phosphorus building gypsum composites. Silic. Bull..

[30] Zhu C., Zhang J., Peng J., Cao W., Liu J. (2018). Physical and mechanical properties of gypsum-based composites reinforced with PVA and PP fibers. Constr. Build. Mater..

[31] Xu S.L. (2009). Direct tensile test of ultra-high toughness cementitious composites. J. Civ. Eng..

[32] Pang Z.M., Cong L., Liu J.X. (2012). Experimental study on tensile properties of strain hardening cementitious composites (SHCC). Ind. Build..

[33] Zhang J., Ju X.C., Guo Z.L. (2009). Effect of PVA fiber diameter on tensile properties of cementitious composites. J. Build. Mater..

[34] Li Y., Liu Z.J., Liang X.W. (2013). Uniaxial Tensile Properties of High Performance PVA Fiber Reinforced Cementitious Composites. Eng. Mech..

[35] (1999). Determination of Physical Properties of Building Gypsum Paste.

[36] (1999). Determination of Mechanical Properties of Building Gypsum.

[37] Zhao B.Y., Zhao Z.M., Quan S.C., Zhang Y., Wu L., Li D. (2019). Effect of carbon fiber content on mechanical properties of phosphorus building gypsum. Non-Met. Mine.

[38] Wu L., Zhao Z.M., Tian R., Wu B., He J.Y., Liu Y., Dai H.J. (2018). Study on strength of cut polypropylene fiber reinforced phosphorus building gypsum. Non-Met. Mine.

[39] Araya-Letelier G., Antico F.C., Burbano-Garcia C., Concha-Riedel J., Norambuena-Contreras J., Concha J., Flores E.S. (2021). Experimental evaluation of adobe mixtures reinforced with jute fibers. Constr. Build. Mater..

[40] Zhang P., Wang L., Zheng Y.X. (2021). Study on flexural toughness of PVA fiber reinforced nano-SiO_2_ cement-based composites. New Build. Mater..

[41] Cao W.X., Peng J.H., Yi W., Zhu C. (2018). Study on the effect of PVA fibers with different lengths on the properties of building gypsum. Non-Met. Miner..

